# YOLO-PBESW: A Lightweight Deep Learning Model for the Efficient Identification of Indomethacin Crystal Morphologies in Microfluidic Droplets

**DOI:** 10.3390/mi15091136

**Published:** 2024-09-06

**Authors:** Jiehan Wei, Jianye Liang, Jun Song, Peipei Zhou

**Affiliations:** School of Mechatronic Engineering, Guangdong Polytechnic Normal University, Guangzhou 510665, China; jiehanwei@gpnu.edu.cn (J.W.); cabyunsatin@outlook.com (J.L.); songjun@gpnu.edu.cn (J.S.)

**Keywords:** microfluidic droplets, crystal morphologies, object detection, deep learning, improved-YOLOv8

## Abstract

Crystallization is important to the pharmaceutical, the chemical, and the materials fields, where the morphology of crystals is one of the key factors affecting the quality of crystallization. High-throughput screening based on microfluidic droplets is a potent technique to accelerate the discovery and development of new crystal morphologies with active pharmaceutical ingredients. However, massive crystal morphologies’ datum needs to be identified completely and accurately, which is time-consuming and labor-intensive. Therefore, effective morphologies’ detection and small-target tracking are essential for high-efficiency experiments. In this paper, a new improved algorithm YOLOv8 (YOLO-PBESW) for detecting indomethacin crystals with different morphologies is proposed. We enhanced its capability in detecting small targets through the integration of a high-resolution feature layer P2, and the adoption of a BiFPN structure. Additionally, in this paper, adding the EMA mechanism before the P2 detection head was implemented to improve network attention towards global features. Furthermore, we utilized SimSPPF to replace SPPF to mitigate computational costs and reduce inference time. Lastly, the CIoU loss function was substituted with WIoUv3 to improve detection performance. The experimental findings indicate that the enhanced YOLOv8 model attained advancements, achieving AP metrics of 93.3%, 77.6%, 80.2%, and 99.5% for crystal wire, crystal rod, crystal sheet, and jelly-like phases, respectively. The model also achieved a precision of 85.2%, a recall of 83.8%, and an F1 score of 84.5%, with a mAP of 87.6%. In terms of computational efficiency, the model’s dimensions and operational efficiency are reported as 5.46 MB, and it took 12.89 ms to process each image with a speed of 77.52 FPS. Compared with state-of-the-art lightweight small object detection models such as the FFCA-YOLO series, our proposed YOLO-PBESW model achieved improvements in detecting indomethacin crystal morphologies, particularly for crystal sheets and crystal rods. The model demonstrated AP values that exceeded L-FFCA-YOLO by 7.4% for crystal sheets and 3.9% for crystal rods, while also delivering a superior F1-score. Furthermore, YOLO-PBESW maintained a lower computational complexity, with parameters of only 11.8 GFLOPs and 2.65 M, and achieved a higher FPS. These outcomes collectively demonstrate that our method achieved a balance between precision and computational speed.

## 1. Introduction

Crystallization is an operation employed within the pharmaceutical, chemical, materials, and food industries for the targeted formation, purification, and isolation of crystalline organic compounds. The quality of the crystallization is directly linked to pharmacological and therapeutic effectiveness. One of the most important characteristics for evaluating the quality of a crystal is its morphology [1,2]. Additionally, pharmaceutical crystallization also plays a crucial role in downstream processing like the filtration, the drying, and the milling of active pharmaceutical ingredients (APIs) [3,4,5]. However, the inherent crystal morphology of many drugs can significantly hinder these processes. To address this challenge, crystal engineering strategies have been employed to manipulate crystal growth and to achieve desirable morphologies. Antisolvent crystallization is a commonly used method for purifying active pharmaceutical ingredients (APIs) [6,7]. This technology usually involves targeting specific intermolecular interactions through the meticulous selection of solvents and the inclusion of tailored additives. The concentration ratios of the drug solvent to the antisolvent can significantly affect the crystal morphology, which in turn influences the concentration of APIs and ultimately impacts the clinical efficacy of the drug. Kim et al. [8] investigated the crystallization of indomethacin using supercritical carbon dioxide and aqueous antisolvent methods, exploring the impact of experimental conditions and habit-modifying agents on crystal size, morphology, and polymorphic form. Their experiments concluded that variations in drug concentration and changes in the mixing rate of the drug and antisolvent can affect the size of the indomethacin crystals, while using different antisolvents results in distinct crystal morphologies, including crystal sheets, crystal rods, and crystal wires. Additionally, precise control of the experimental parameters during crystallization is crucial. Although effective, this traditional approach is inherently empirical, relying on the scientist’s expertise and often requiring extensive trial-and-error experimentation. This can create a significant bottleneck in terms of time, resource consumption, and overall efficiency [9]. Droplet microfluidic technologies can precisely control experimental conditions, reduce operation time, and conduct a substantial volume of crystallization experiments only using a minimal quantity of APIs [10,11,12]. Sun et al. [13] developed a droplet-based system for protein crystallization, which enabled automated sample introduction and the generation of droplets at an ultra-high throughput of up to 6000 droplets per hour. Yadavali et al. [14] developed a microfluidic droplet platform that efficiently generated large quantities of polymer microparticles, achieving a production rate of 227 g/h. This system significantly enhances production rates, offering a scalable solution for pharmaceutical applications. Fortt et al. [15] produced spherical crystalline particles of the antiretroviral HIV API cabotegravir via solvent extraction on droplet microfluidic devices. Parallelization led to a 100-fold increase in throughput over single-channel devices, with the resulting drug excipient demonstrating stability during downstream processing. However, the ultra-high throughput of droplet microfluid generates extensive datasets covering many small-object crystals which also obscure each other. Faced with this situation, it is hard for humans to quickly identify different crystal morphologies and it is expensive to utilize automatic screening platforms (such as CrystalQuick X plates from Greiner Bio-One or In Situ-1 plates from MiTeGen, which also come with high costs and require specialized hardware like translational stages for crystal alignment) [16,17,18]. These problems lead to a lack of high-throughput-characterization approaches, which results in the limitation of production rates [19].

Recently, both the advent and the progression of artificial intelligence have facilitated some methods based on deep learning for object detection and classification, thereby making it possible to achieve the fast and accurate detection of crystal morphologies [20]. Scholars from Thailand utilized the VGG16 model to identify sugar crystals [21]. Scholars from Princess Nourah Bint Abdulrahman University combined a squeeze-excitation (SQE) with a dense neural network (DCNN) to achieve high accuracy and efficiency in defect detection in silicon nitride crystal structures [22]. Yann et al. designed the CrystalNet, utilizing a convolutional neural network (CNN) which was trained on a dataset comprising 163,894 high-resolution, grayscale labeled images derived from protein crystallization trials conducted on 1536-well plates [23]. This model achieved an accuracy of 0.908 and an impressive area under the receiver operating characteristic curve (AUC-ROC) of 0.9903 for the classification of crystal classes. Manee et al. modified RetinaNet, designing a novel deep learning network to solve the problem of crystal detection in high-density solute [24].

This paper builds on this work [11] by further examining and employing indomethacin, a nonsteroidal anti-inflammatory drug [25], as a research subject. We used droplet microfluidic technologies to obtain different morphologies of indomethacin, including crystal wire, crystal sheet, crystal rod, and jelly-like phase. The impact of crystal morphology on the properties of indomethacin is substantial. Different crystal forms and shapes can greatly influence a drug’s physical and chemical properties, including its solubility, its dissolution rate, and ultimately its bioavailability and efficacy [26]. The presence of jelly-like substances during the crystallization process can significantly reduce the crystallization rate of the active drug components, hindering the production of high-quality crystals [27]. Indomethacin’s polymorphism is complex, but the α-form and the γ-form are the most produced and useful forms [28]. Crystal wire corresponds to the γ-form of indomethacin, whereas crystal rod and crystal sheet belong to the α-form. Based on their different physical and chemical properties, these crystal forms can be developed into various pharmaceutical dosage forms, such as tablets and capsules [8,29,30]. Additionally, the formation of crystal wire, crystal sheet, and crystal rod requires different drug concentrations in the solvent, as well as varying types and ratios of antisolvents, which in turn results in varying drug concentrations. Manual identification of these different crystal forms is inefficient, and any errors in identification can lead to the waste of both the active pharmaceutical ingredients and the solvents. To solve the problems of slow manual detection and classification of different indomethacin crystal morphologies and the poor recognition of small target crystals, we proposed an improved detection algorithm (YOLO-PBESW) of crystal morphologies based on YOLOv8. Firstly, the original YOLOv8 only has three feature layers—P3, P4, and P5—which are inadequate for the detection of the small target crystals. To enhance the detection of small target crystals, we integrated the high-resolution feature layer P2, improving the acquisition of more shallow details and location information. Secondly, PANet architecture is adopted in the neck of YOLOv8. We utilized the BiFPN [31] for multi-scale feature fusion to improve the detection performance and to reduce the computation cost. This paper proposed the incorporation of an EMA mechanism within the architecture. Furthermore, the introduction of the wise-IoU loss function has enhanced the model’s robustness and generalization. This improvement was particularly crucial when the dataset covered low-quality examples.

This paper’s contributions can be summarized as follows:

1. Incorporating the high-resolution feature layer P2 into the architecture to preserve greater detail and location information that enhance the accuracy of the detection of small target crystals;
2. Adopting the methodology of BiFPNs to design a structure that combines features from different scales in place of the original PANet;
3. Adding the EMA mechanism to enhance feature fusion abilities;
4. Utilizing WIoU_v3 as an enhanced loss function to improve the accuracy and generalization of predicted bounding boxes.

## 2. Related Work

The task of object detection is not only the classification of objects but also the accurate localization of these objects within imagery [32]. There are two main periods in object detection: the conventional object detection era (1998–2014) and the period of object detection based on deep learning (2014–now). In the period of conventional object detection, some methods like scale invariant feature transform (SIFT) [33] were used; SIFT was an image descriptor used for object detection and image matching. Histograms of Oriented Gradients (HOG) [34] were also used, which worked by computing and compiling histograms of oriented gradients in localized portions of images which had limitations such as complex backgrounds, multi-scale objects, and small targets. Feng et al. [35] utilized support vector machines (SVM) to classify crystal, leading to a high false-positive rate due to low precision. With the advancement of deep learning and computational capabilities, the new era of object detection has made huge progress [36]. The main stream of object detection based on deep learning can be divided into two category: two-stage algorithms and one-stage algorithms [37].

Two-stage algorithms like R-CNN [38], Fast R-CNN [39], and Faster R-CNN [40] typically follow a two-stage process. In the first stage, the algorithm generates a range of region proposals (RPN) that efficiently identify and extract image regions that contain objects of interest. In the second stage, the algorithm utilizes convolutional networks to classify and regress these proposals. Gao et al. [41] utilized the mask regional convolutional neural network (Mask R-CNN) to validly achieve the segmentation and categorization of two distinct morphological classes of LGA crystals. But the processing speed was only 10 frames per second. Su et al. [11] developed an integrated system that merges a hydrogel droplet-based platform with the Faster R-CNN deep learning algorithm to facilitate the high-throughput screening of antisolvent crystallization conditions for active pharmaceutical ingredients (APIs). But they did not modify and optimize the network architecture, resulting in limitations in both accuracy and detection speed.

Although these algorithms have advantages in detection accuracy, they are often complex and have limitations in detection speed. One-stage algorithms are different to two-stage algorithms. They directly compute the class probability and spatial coordinates of objects, getting the final detection outcomes only through a singular stage. This methodology reduces computation and enhances detection speed but has disadvantages for accuracy. The representation of one-stage algorithms includes the YOLO series and SSD [42]. Jiang et al. [43] proposed a method based on YOLOv4 to reduce the labeling effort and computation load of real-time image segmentation. They used rectangular boxes for object detection to evaluate the characteristic size of crystals, enabling real-time implementation. Fan et al. [44] utilized the YOLOv5 algorithm to detect the scintillation crystals, and the recognition rate and confidence can reach 98% and 80%, respectively. Since our detection task needs to meet the demand for speed and accuracy, we recommended the YOLOv8, the state-of-the-art algorithm of the YOLO series, as the base model. We modified and improved its architecture to achieve the fast and precise detection of different indomethacin crystal morphologies.

## 3. Methodology

### 3.1. Improved YOLOv8

**Figure 1:**
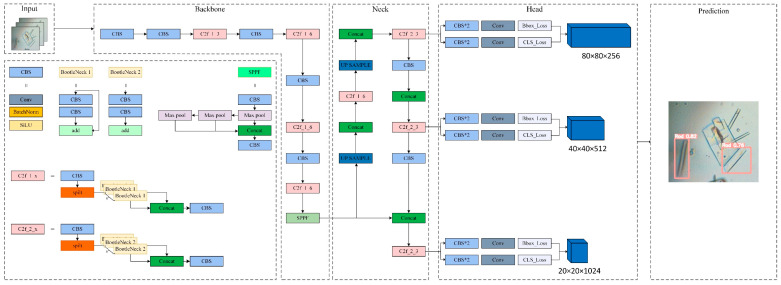
YOLOv8 network structure.

YOLO (you only look once) is one of the single-stage detection algorithms known for its fast speed and high accuracy. The latest iteration, YOLOv8, introduced by Ultralytics in 2023, offers a range of configurations tailored for different performance and resource requirements. There are several reasons for our choice of YOLOv8 as the base model. Firstly, compared with YOLOv5 and YOLOv7 [45], YOLOv8 demonstrates notable enhancements in mean Average Precision (mAP), parameter count, and Floating Point Operation per second (FLOPs) outcome, when all models were tested on the COCO dataset [46]. Secondly, the limitations of YOLOv5 include challenges in detecting small targets and a requirement for enhancements in dense-target detection. Additionally, the performance of YOLOv7 is constrained by the quality and quantity of training data, the structure of the model, and the hyperparameter settings during training [47]. Thirdly, there exists a large and active user community of YOLOv8 to foster the development and dissemination of readily available implementation resources. The YOLOv8 is provided in a number of different variants, including YOLOv8n, YOLOv8s, YOLOv8m, YOLOv8l, and YOLOv8x. Each of these is designed to handle different detection tasks. The objective of this paper is to design and implement a more efficient algorithm for the detection of crystal morphologies, with the aim of improving high-throughput characterization approaches. Accordingly, the YOLOv8 algorithm’s nano model has been selected as the original model, due to its recognition speed, and its structure has been shown in Figure 1.

**Figure 2:**
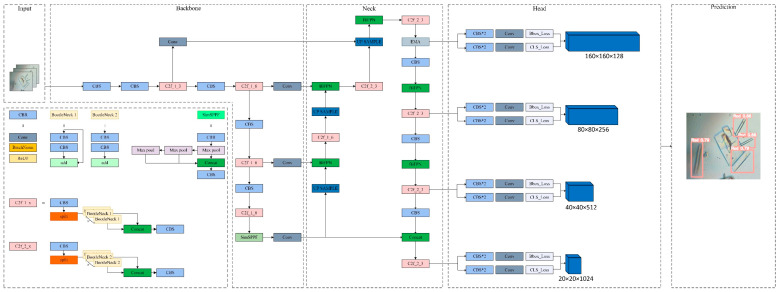
YOLOv8-PBEWS. Its improvement encompassed an additional 160 × 160 detection layer with EMA mechanism, the integration of BiFPN between the backbone and neck, the replacement of the original SPPF with SimSPPF, and the substitution of the loss function with WIou.

The architectural design of the YOLOv8 model encompasses three principal components: the backbone, the neck, and the head. To enhance the quality of detection, we introduced structural improvements to these three key parts. The model’s structure has been illustrated in Figure 2. Firstly, we added a high-resolution feature layer P2 to acquire more location information, which proved advantageous in the detection of small targets. Secondly, we utilized the methodology of BiFPNs to replace the PANet structure, which resulted in a reduction in computational complexity and an improvement in detection accuracy. Thirdly, we added the EMA mechanism before the detection head to improve the detection performance while keeping the model lightweight. Additionally, we substituted the SimSPPF module with SPPF to reduce the inference time. Finally, we chose the WIoUv3 loss function to replace the CIoU loss function. The specific modifications are described as follows:

#### 3.1.1. High-Resolution Feature Layer P2

**Figure 3:**
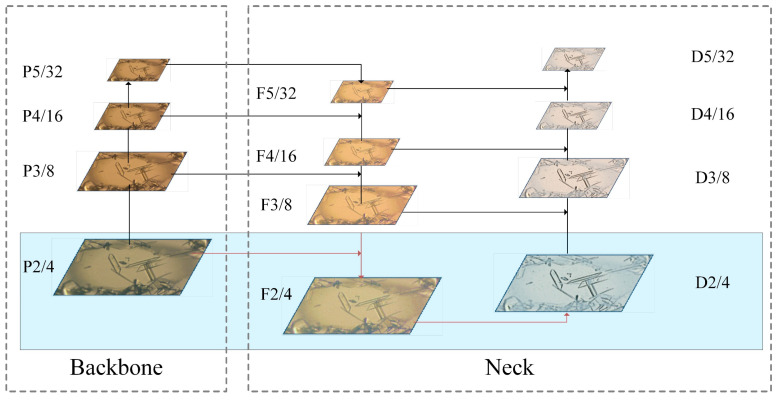
Structure with high-resolution detection layer P2. Red lines indicate newly added P2 layer.

Given the presence of some small crystal targets in our dataset and the large downsampling of YOLOv8, acquiring feature information for small targets which form deeper feature maps poses a challenge. There are three instances of downsampling in the YOLOv8 algorithm: 32-time, 16-time, and 8-time. With larger downsampling, the stronger semantic representation of the features is extracted. But it results in more location-information loss which is not of benefit for the detection of small targets. For example, a downsampling of 8 will generate an 80 × 80 detection scale, while the sensory field from the detection of each grid is 8 × 8. If the target has heights and widths less than 8 pixels, the original YOLOv8 algorithm will struggle to discern it. The features derived from shallower layers with less downsampling retain more locational information, which is advantageous for the detection of small targets. Since there are a large number of small targets in our crystal datasets, a high-resolution feature map with a 4-time downsampling with a detection scale of 160 × 160 was added to the backbone. Its structure is displayed in Figure 3. Incorporating the 160 × 160 scale layer facilitates the propagation of small-crystal information throughout the downsampling, thereby reinforcing the model’s capacity for feature fusion and enhancing the precision of small-target detection. Furthermore, the introduction of an additional detection head extends the detection range for crystals of small size. The enhancement in detection accuracy and range enables the network to more precisely recognize crystal morphologies within the droplet.

#### 3.1.2. BiFPN

**Figure 4:**
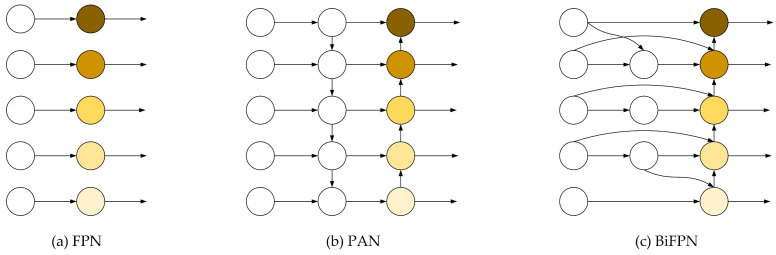
Feature network design. (**a**) a FPN, (**b**) a PANet, which adds a bottom-up pathway on top of a FPN, and (**c**) a BiFPN, which reduces the number of nodes while achieving cross-scale feature fusion.

Feature pyramid networks (FPNs) employ a top-down methodology, as shown in Figure 4a, enhancing the resolution of the features by upsampling the coarse spatial information present at lower levels and merging it with the semantically richer feature maps at elevated pyramid levels. Path Aggregation Networks (PANs) build upon FPNs by incorporating a complementary bottom-up pathway, as shown in Figure 4b. For example, there is a list of multi-scale features $\vec{P^{in}}=(P_{l1}^{in},P_{l2}^{in},P_{l3}^{in},\ldots )$; here, $\vec{P^{in}}$ denotes the feature at level $l_{i}$. Additionally, feature fusion generates a sequence of intermediate features $\vec{P^{td}}=(P_{l1}^{td},P_{l2}^{td},P_{l3}^{td},\ldots )$. PAN adopts a simple summation strategy for the integration of multi-scale features.

(1) $$P_{li}^{td}=Conv(P_{li}^{in}+Resize(P_{li+1}^{td})$$

(2) $$P_{li}^{out}=Conv(P_{li}^{out}+Resize(P_{li+1}^{out})$$

where Resize represents an upsampling or downsampling operation, Conv represents a convolution operation, and $P^{out}$ denotes the output feature at level $l_{i}$.

This addition facilitates the propagation of precise localization information from the lower levels to the higher levels of the network. The original neck network of YOLOv8 employs a combination of FPNs and PANs that enables the fusion of features across various layers. But Li et al. [48] found that this structure may filter out some essential feature information and result in a large computational cost. Tan et al. [31] proposed the bi-directional feature pyramid network (BiFPN), as displayed in Figure 4c. The BiFPN simplifies the network by removing nodes with only one input edge, as these nodes contribute less to the process of feature fusion. By focusing on nodes that integrate multiple features, the BiFPN reduces computational complexity while still effectively capturing and combining important features compared with FPNs and PANs. It has two main directions to achieve cross-layer information transfer and feature fusion: upward convergence from lower feature layers and downward convergence from higher feature layers. Combining features from various layers and assigning appropriate weights means faster multi-scale target detection. For example, two fused features at level 4 for BiFPN are as follows:

(3) $$P_{4}^{td}=Conv(\frac{w_{1}\cdot P_{4}^{in}+w_{2}\cdot Resize(P_{5}^{in})}{w_{1}+w_{2}+\varepsilon })$$

(4) $$P_{4}^{out}=Conv(\frac{w_{1}^{\prime }\cdot P_{4}^{in}+w_{2}^{\prime }\cdot P_{4}^{td}+w_{3}^{\prime }Resize(P_{5}^{in})}{w_{1}^{\prime }+w_{2}^{\prime }+w_{3}^{\prime }+\varepsilon })$$

through applying ReLU after each $w_{i}$ to ensure $w_{i}>0\;(i=1,\;2,\;3,\;\ldots )$. The value ε is to prevent numerical instability. At level 4 on the top-down pathway, $P_{4}^{td}$ serves as the intermediate feature, and $P_{4}^{out}$ is the output feature at level 4 on the bottom-up pathway.

In hydrogel droplets, crystal sheets and crystal rods have identical morphologies and sizes. It is difficult for the PAN of the original YOLOv8 to identify them. This paper utilized the methodology of BiFPNs to fuse cross-level feature information for improving the detection accuracy of crystal rods and crystal sheets.

#### 3.1.3. EMA Mechanism

**Figure 5:**
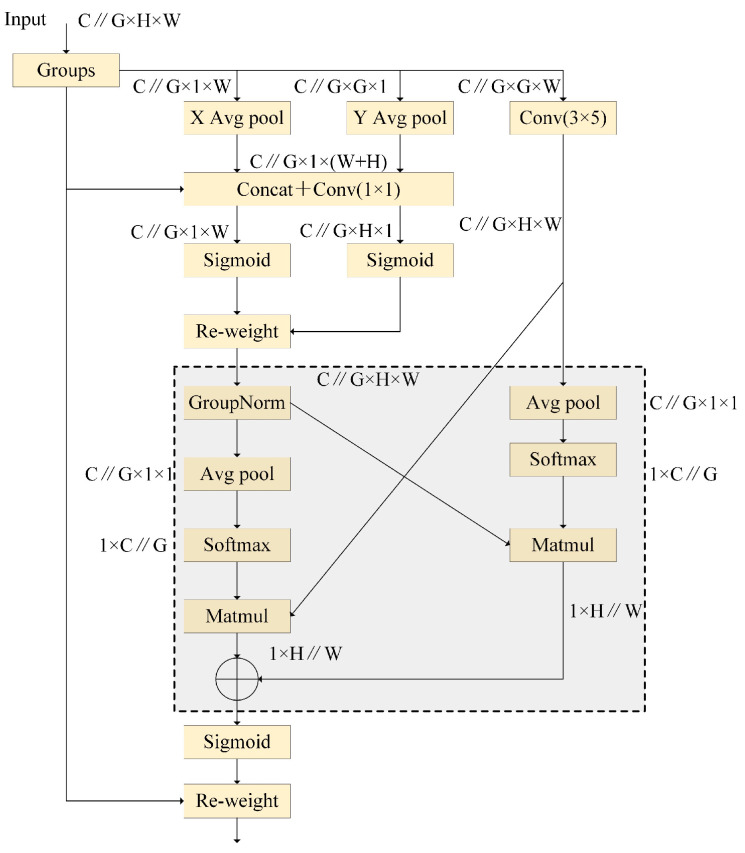
Structure of EMA.

The EMA [49] mechanism is an innovative and effective multiscale attention module. It is unlike previous attention mechanisms, such as the coordinate attention mechanism (CA) [50], which incorporates positional information to enhance spatial feature extraction but has limitations in capturing all of the critical information facing complex spatial information, and the channel attention mechanism, which refines feature representations by exploiting inter-channel relationships and amplifying informative channels but fails to address the significance of feature information across various spatial scales. The EMA mechanism incorporates the ability to analyze features across a spectrum of scales, ranging from small to large scale, that can achieve better performance in intricate detection environments. The structure of the EMA mechanism is shown in Figure 5. Given any input feature tensor $X\in R^{C\times H\times W}$, EMA partitions X across the channel dimension, resulting in G sub-features learning different semantics. To extract attention weight descriptors from the segmented feature groups, EMA leverages three parallel processing pathways. Two of these pathways operate a 1×1 convolution branch, while the third pathway utilizes a 3×3 convolution branch. This architecture effectively captures intricate dependencies and minimizes the computational expense. Within the 1×1 convolution branch, two independent 1D global average pooling operations are strategically employed. These operations have the capability of encoding channel-wise information across both spatial dimensions (height and width) separately. The 3×3 convolution branch utilizes a single 3×3 kernel to capture multi-scale feature representations. Following the application of two independent 1D global average pooling operations, EMA incorporates a processing methodology from CA, employing concatenation to two encoded features along the image-height dimension and making it share the same 1×1 convolution. After the convolution, the output is factorized into two vectors. To ensure compatibility with the subsequent linear convolutions, both vectors are passed through separate non-linear sigmoid activation functions. Then, the “re-weight” operation mixes features from various scales to improve or suppress features through the application of an attention map, which is deduced from the input feature map onto the original map. After that, as shown in Equation (5), 2D global average pooling is used to encode the global spatial information of the 1×1 branch’s outputs. The outputs of the smallest branch will be converted to the shape $R_{1}^{\left(1\times C∥G\right)}\times R_{3}^{\left(C∥G\times HW\right)}$. To ensure efficient computation, EMA uses the softmax function for 2D Gussian maps at the outputs of 2D global average pooling to adjust for linear transformations. The first spatial attention map was derived by multiplying the outputs of the above parallel processing with matrix dot-product operations. Furthermore, the same 2D global average pooling was used to encode the global spatial information of the 3 × 3 branch’s outputs, and the 1 × 1 branch was converted to the shape $R_{3}^{\left(1\times C∥G\right)}\times R_{1}^{\left(C∥G\times HW\right)}$. Following this, the second spatial attention map was derived, which preserved precise spatial positional information. In the end, EMA combines the spatial attention weight values, which are derived from the output feature maps using the sigmoid function. This approach focuses on understanding the relationship between each pair of pixels and emphasizes global contexts for all pixels.

(5) $$Z_{c}=\frac{c1}{H\times W}\sum_{j}^{H}\sum_{i}^{W}x_{c}(i,j)$$

#### 3.1.4. WISE-IoU Function

In the domain of object detection, the loss function plays a crucial role in determining the overall performance of the model. Selecting an appropriate loss function can significantly enhance the accuracy and robustness of the bounding box predictions, thereby improving the model’s ability to precisely localize and classify objects within an image.

Different from YOLOv5, which only uses CIoU loss, YOLOv8 utilizes two kinds of regression loss: CIoU loss and DFL (distribution focal loss) [51]. The equations of CIoU are as follows:

(6) $$Loss_{CIoU}=1-IoU+\frac{\rho^{2}(b,b^{gt})}{c^{2}})+\alpha v;$$

(7) $$\alpha =\frac{v}{(1-Iou)+v};$$

(8) $$v=\frac{4}{\pi^{2}}{\left(tanh^{-1}\frac{w_{y}}{h_{y}}-tanh^{-1}\frac{w_{x}}{h_{x}}\right)}^{2},$$

where α denotes the coefficient employed to balance competing objectives and the parameter v assesses the consistency of the aspect ratio. The centers of the predicted and labelled boxes are represented by b and $b^{gt}$, respectively. ρ denotes the Euclidean distance between the two center points, while c represents the length of the diagonal line of the minimum outer rectangle that contains both the predicted box and the labeled box.

While the CIoU loss function that is applied by YOLOv8 offers significant improvements over conventional IoU loss by effectively addressing challenges such as bounding box offset and aspect ratio imbalances in object detection frameworks, its primary utility lies in improving the fitting precision of bounding box regression. But the low-quality data within the dataset will result in model overfitting as bounding box regression is overly emphasized for such low-quality examples. This overfitting can subsequently diminish the overall detection performance of the model. Since our dataset includes some blur and small crystals, CIoU’s dependence on distance and aspect ratio metrics may unfairly penalize these lower-quality examples. Wise-IoU (WIoU) [52] incorporates weights for the region between predicted bounding boxes and ground truth boxes to solve this problem. There are three versions of WIoU. WIoU_v1 employs an attention mechanism to construct the bounding box loss, focusing on crucial areas within the box to optimize localization accuracy:

(9) $$L_{IoU}=1-IoU;$$

(10) $$R_{WIoU}=exp\left[\frac{(x-x_{gt})+{\left(y-y_{gt}\right)}^{2}}{{\left(w_{g}^{2}+H_{g}^{2}\right)}^{*}}\right];$$

(11) $$L_{WIoU\_v1}=R_{WIoU}L_{IoU},$$

where the loss of IoU, denoted as $L_{IoU}$, is defined as the complement of the IoU between the predicted bounding boxes and the ground truth. It focuses on the quality of the overlap. x and y are the expected bounding box’s center coordinates. The width and height of the minimum bounding box are represented by $w_{g}$ and $H_{g}$, respectively. $w_{gt}$ and $H_{gt}$ separately represent the coordinate of the ground truth bounding box’s center. The $R_{WIoU}$ quantifies the normalized distance between the center points of the predicted and the ground truth bounding boxes.

WIoU_v2 introduces a monotonic static focus mechanism (FM), as shown in Equation (12):

(12) $$L_{WIoU\_v2}={\left(\frac{L_{IoU}^{*}}{\bar{L_{IoU}}}\right)}^{\gamma }L_{WIoU\_v1}(\gamma >0)$$

where $\bar{L_{IoU}}$ represents the exponential running average with momentum. To solve the problem of slow convergence in the late stages of training, the gradient gain $\frac{L_{IoU}^{*}}{\bar{L_{IoU}}}$ keeps a high level overall.

WIoU_v3 employs a gradient gain as the focusing coefficient, utilizing a nonmonotonic dynamic focusing mechanism to allocate the loss function strategically. This approach ensures that the model prioritizes samples which are challenging to match accurately with the target, thereby significantly enhancing the detection accuracy and robustness. The equations are as follows:

(13) $$\beta ={\left(\frac{L_{IoU}^{*}}{\bar{L_{IoU}}}\right)}^{\gamma };$$

(14) $$r=\frac{\beta }{\delta \alpha^{\beta -\delta }};$$

(15) $$WIoU\_v3=rL_{WIoU\_v3},$$

where β represents the mapping of outlier degrees. r is the gradient gain, and the hyper-parameters α and δ adjust it.

Compared with WIoU_v1 and WIoU_v2, the $\bar{L_{IoU}}$ is dynamic, and the standard for demarcating the quality of anchor boxes is similarly dynamic, so WIoU_v3 can employ a gradient gain allocation strategy that aligns optimally with the prevailing conditions at any given moment. For these reasons, we adopted WIoU_v3 to replace the original CIoU, setting *α* value to 1.9 and the δ value to 3. This configuration is designed to allocate smaller gradient gains to lower-quality anchor boxes, thus enhancing the effectiveness of the bounding box loss function.

#### 3.1.5. SimSPPF

**Figure 6:**
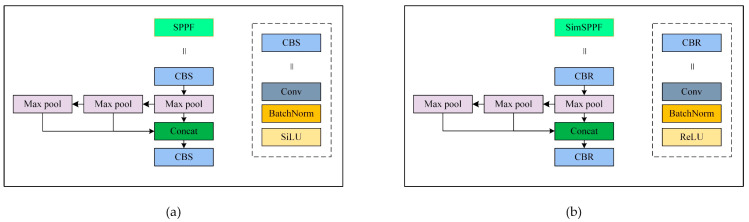
Comparison of the SPPF module and the SimSPPF module: (**a**) represents SPPF, CBS in the SPPF module refers to a sequence of convolution, batch normalization, and SiLU activation; and (**b**) represents SimSPPF, CBR in the SimSPPF module represents a sequence of convolution, batch normalization, and ReLU activation.

For the purpose of improving the speed of detection, we substituted the conventional Spatial Pyramid Pooling Fusion (SPPF) module (as shown in Figure 6a) in the YOLOv8 with the quicker Simple Spatial Pooling Fusion (SimSPPF) module, as shown in Figure 6b. Different from the Shifted Linear Unit (SiLU) activation function that is adopted by SPPF, SimSPPF uses the Rectified Linear Unit (ReLU) activation function that mitigates the issue of vanishing gradients and facilitates more rapid convergence. Two of these activation functions as shown in Equations (16) and (17):

(16) ReLU:f(x)=x, x>00, x<=0

(17) $$SiLU:\;f(x)=\frac{x^{2}}{1+e^{-x}}$$

## 4. Experiments

### 4.1. Experiments’ Environments and Training Paraments

The configuration of the experimental environment is shown in Table 1. The experiments were performed on a workstation with 128 GB RAM, Intel Xeno Sliver 4210R (10 Cores and 20 Threads), and Nvidia Quadro RTX5000 (16 GB storage). And all the experiments were based on Windows 10, Python 3.8, Pytorch 2.0.1, and CUDA 11.8.

The training setting is shown in Table 2. In the training stage, we employed the Stochastic Gradient Descent (SGD) optimizer with a weight decay and momentum of 0.0005 and 0.937, respectively. The learning rate was set as 0.01. Additionally, the number of training epochs was set to 300 with a batch size of 12, and the input image resolution was set to 640 × 640. If the training data did not improve within 50 epochs, the training process employed early stopping to prevent overfitting. Training was terminated if the performance metric did not improve for 50 epochs. A learning rate warm-up strategy was implemented for the initial 3 epochs.

### 4.2. Datasets

**Figure 7:**
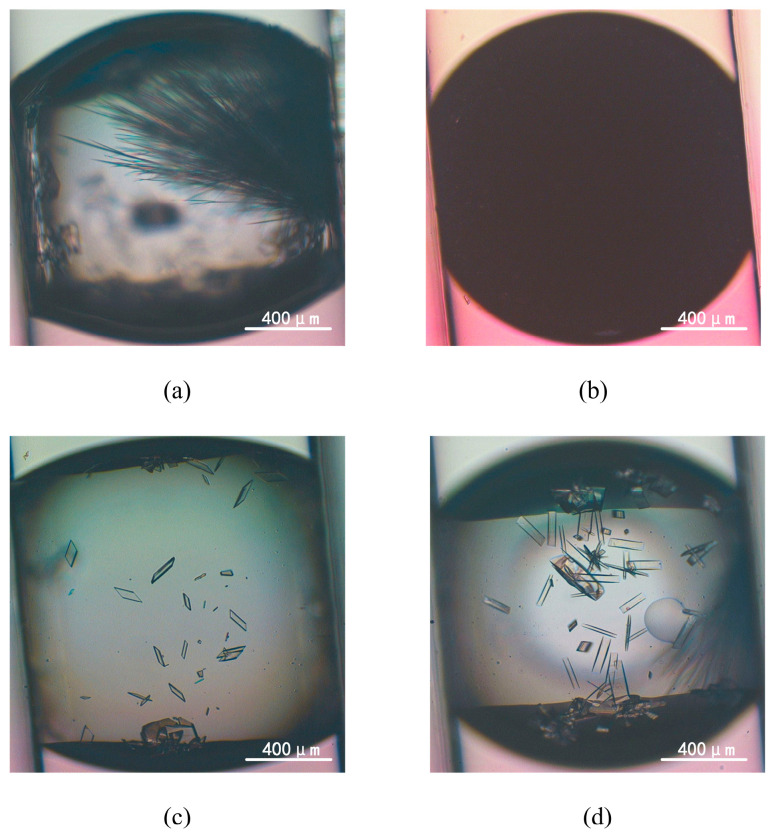
Micrographs depicting three representative Indomethacin crystal morphologies within hydrogel droplets: (**a**) crystal wire, (**c**) crystal sheet, and (**d**) crystal rod, alongside (**b**) amorphous jelly-like phase (JLP). The scale bar at the bottom right corner of each image represents 400 μm.

**Figure 8:**
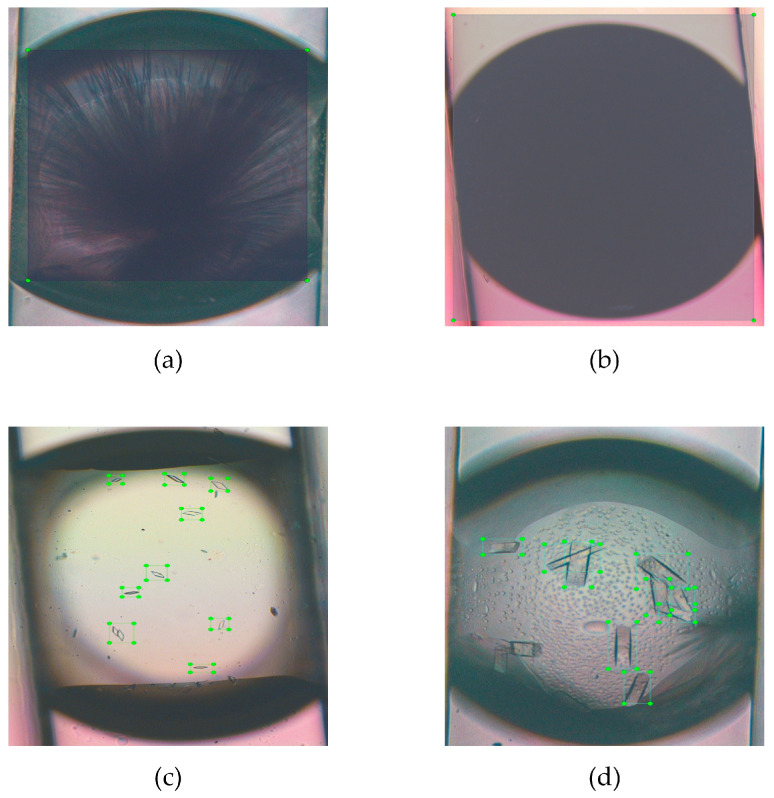
Annotation performed using LabelImg. (**a**) crystal of wire, (**b**) jelly-like phase, (**c**) crystal of sheet, and (**d**) crystal of rod.

**Figure 9:**
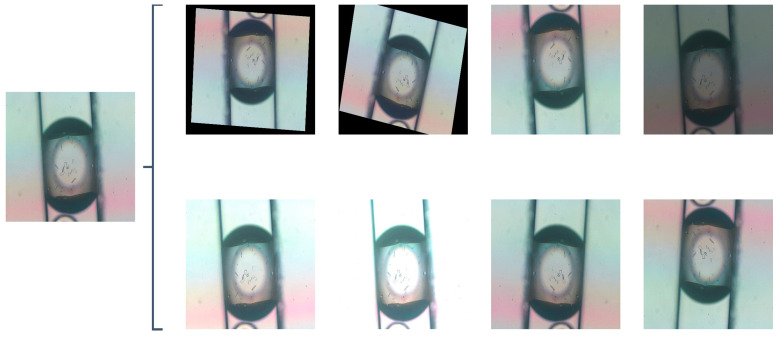
Model-free augmentation.

**Figure 10:**
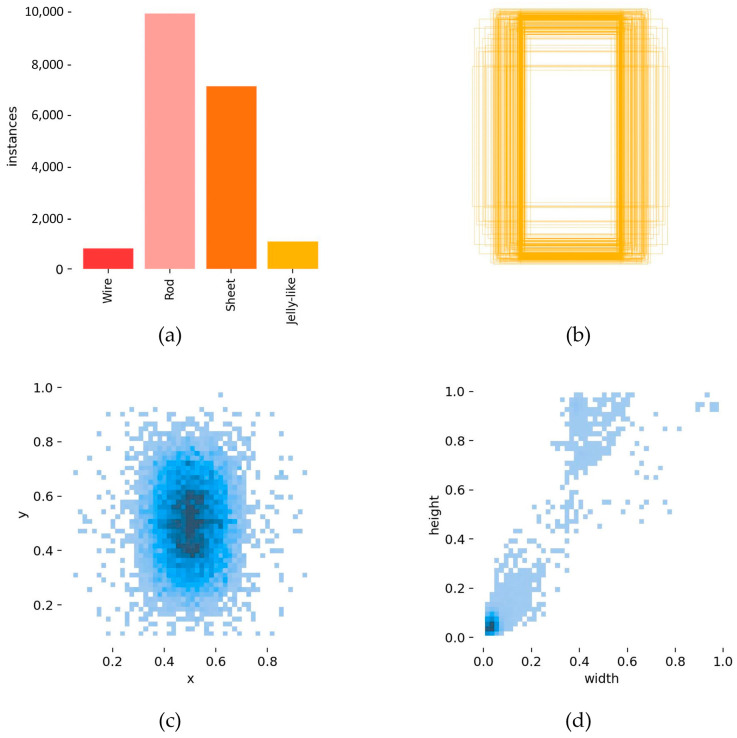
Basic information of training set: (**a**) displays the counts for each detection category; (**b**) depicts the dimensions of each target box, including the total number of boxes and their range of variation; (**c**) reveals the relative position of the target’s center point within the overall image; and (**d**) portrays the aspect ratio of the target’s height to width relative to the entire dataset.

We have developed an advanced high-throughput system employing droplet microfluidic technology to conduct experiments, generating indomethacin crystals with various morphologies within hydrogel droplets. The images of droplets and crystals were taken from fluorescence microscopy (TI-U, Nikon, Tokyo, Japan) with a CCD camera (Digital Sight DS-Fi2, Nikon, Japan) and using imaging software (NIS-Elements BR, Nikon, Japan) to display morphologies of them, as shown in Figure 7. Further details can be found in this work [11]. We utilized the LabelImg annotation tool to delineate the minimum bounding box encompassing crystals and JLP instances. The total number of images was 635, including 191 images of jelly-like phase, 193 images of rod crystals, 127 images of sheet crystals, and 124 images of wire crystals (Figure 8). To enhance the efficiency of the model’s training and evaluation, the raw dataset was randomly divided into training, validation, and testing sets, with a ratio of 6:3:1. Data augmentation in object detection is an effective and efficient method to avoid the model’s low generalization and robustness ability when there is insufficient data [53]. We first preferred model-free augmentation [54] to extend the size of the dataset, including flipping horizontally, flipping vertically, changing light, changing color, rotating, cropping, flipping, and mirroring. Ultimately, we adopted mosaic data augmentation [55], HSV-Hue augmentation, HSV-Saturation augmentation, HSV-value augmentation, image translation, image scaling, and image flipping horizontally to improve the generalization of the model during training time. Mosaic data augmentation randomly combined four training images into a single composite image. Considering the enhanced images that were created, this method was not representative of the true distribution of natural images; mosaic data augmentation was disabled for the last 10 epochs. All of these methods were applied to the training set in Figure 9. Figure 10 demonstrates the fundamental characteristics of the training set, which constitute a portion of the complete dataset, segmented into four primary sections.

### 4.3. Evaluation Metrics

**Figure 11:**
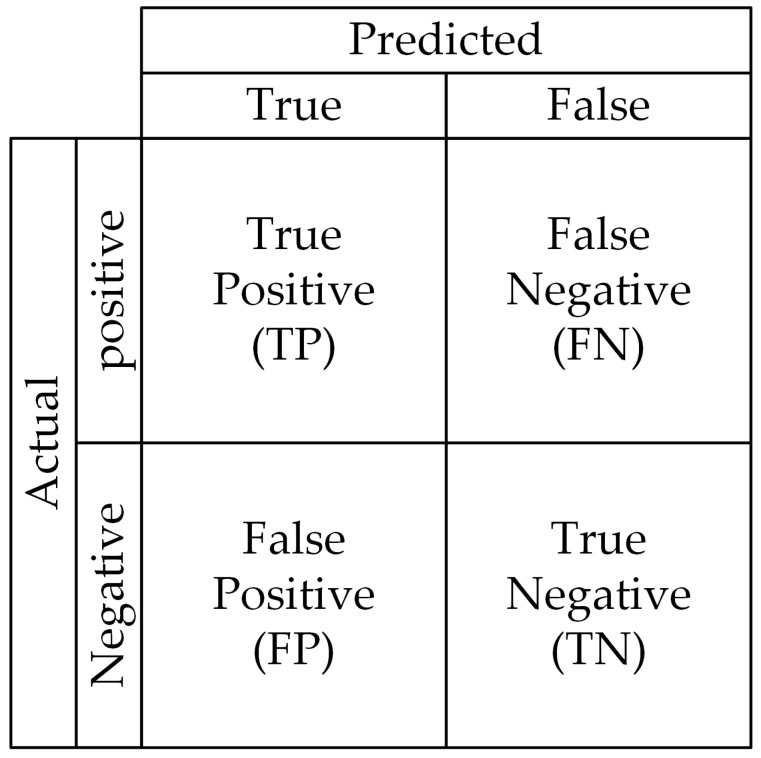
Confusion matrix.

In evaluating the performance of crystal detection, we employed a multifaceted approach by considering various metrics:

Confusion matrix: It provides a visual representation of the classification outcomes for each category, as shown in Figure 11. Each row corresponds to actual categories, while each column represents categories predicted by the model. Values along the diagonal indicate the proportion of instances correctly classified into their respective categories. Based on these definitions, a true positive (TP) is when the model correctly predicts a positive sample, and the actual class is indeed positive. A false negative (FN) occurs when the sample’s true class is positive but the model incorrectly predicts it as negative. A false positive (FP) happens when the sample’s true class is negative yet the model incorrectly identifies it as positive. A true negative (TN) is when the sample’s true class is negative and the model correctly identifies it as negative. The confusion matrix is crucial for calculating various metrics, including precision, recall, and F1-score.

Precision (P): This metric is defined as the proportion of TP outcomes relative to the aggregate number of instances classified as positive, including TP and FP.

(18) $$Precision=\frac{TP}{TP+FP}$$

Recall (R): This metric calculates the ratio of TP to the total of TP and FN.

(19) Recall=TPTP+FN

F1-Score: It represents the harmonic mean of precision and recall.

(20) $$F1\_Score=2\times \frac{P\times R}{P+R}$$

Average precision (AP): AP signifies the mean precision values computed at various recall levels. It also represents the area under the precision–recall curve, providing a comprehensive evaluation of the model’s ability to accurately identify relevant instances across varying recall thresholds. It was calculated by Equation (21), where P indicates precision, and R indicates recall.

(21) $$AP=\int_{0}^{1}P(R)dR$$

Mean Average Precision (mAP): mAP can be calculated by dividing the AP by the total number of classes (num_classes), as the following equation shows:

(22) $$mAP=\frac{AP}{num\_calsses}$$

## 5. Results and Discussion

### 5.1. Experimental Analysis

#### 5.1.1. Confusion Matrix

**Figure 12:**
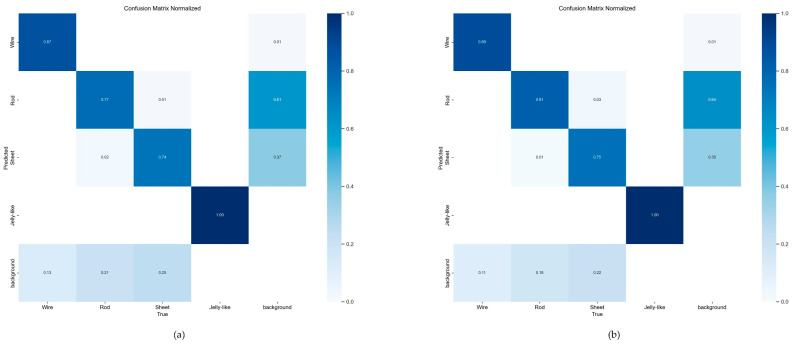
(**a**) Confusion matrix of YOLOv8n and (**b**) confusion matrix of YOLO-PBESW. In each matrix, the rows represent the actual classes, while the columns represent the predicted classes. The diagonal elements indicate the correct classifications, with darker shades representing higher accuracy.

The confusion matrices for YOLOv8n and YOLO-PBESW are presented in Figure 12. Firstly, the YOLO-PBESW demonstrated improved recognition accuracy of crystal wire, crystal rod, and crystal sheet, with respective increases of 2%, 4%, and 1%. Secondly, the misclassification rate of crystal wire, crystal rod, and crystal sheet as background decreased from 13%, 21%, and 25% to 11%, 18%, and 22%, respectively.

#### 5.1.2. P–R Curve

**Figure 13:**
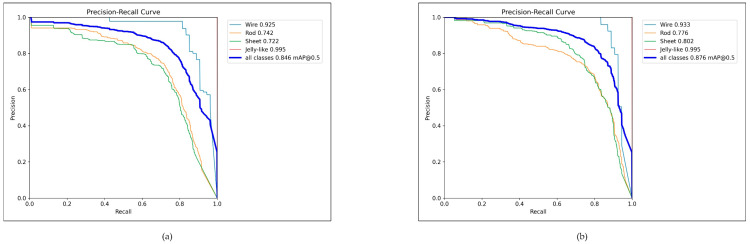
P–R curves: (**a**) depicts the P–R curve of YOLOv8n and (**b**) depicts the P–R curve of YOLO-PBESW.

Figure 13 demonstrates the P–R curve graphs of YOLOv8n and YOLO-PBESW. It is obvious that the area enclosed by each curve of our method is significantly larger than the original YOLOv8n, which indicates that our methods achieved a significant improvement in detection.

#### 5.1.3. Detection

**Figure 14:**
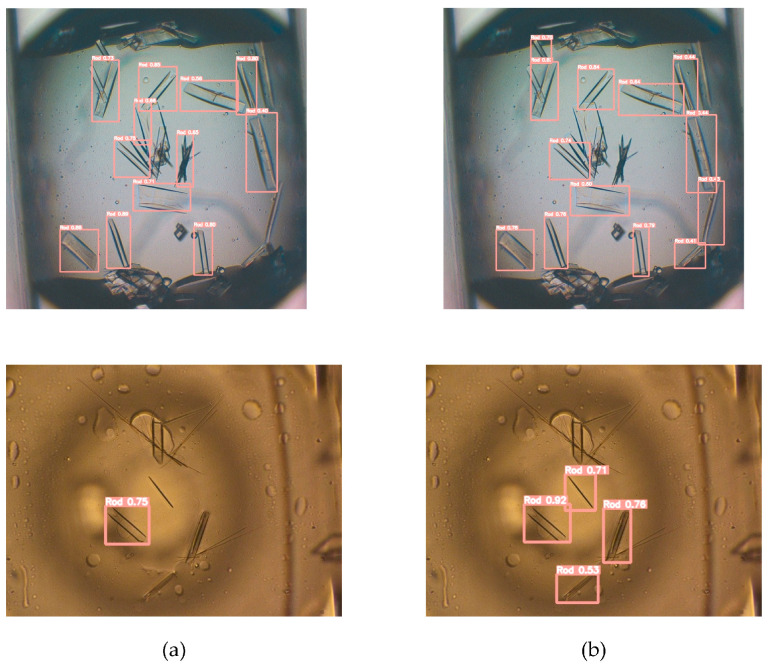
Comparison of detection: (**a**) represents YOLOv8’s detection result and (**b**) represents YOLO-PBESW’s detection result.

Figure 14 shows two sets of comparisons of YOLOv8n and YOLO-PBEWS. In scenarios where the images exhibited intricate backgrounds, particularly in instances of blurred imagery with overlapping crystals of varied types, YOLOv8n often exhibited a tendency to omit detection results. This comparison reveals that our method exhibited superior performance in detecting crystals.

### 5.2. Ablation Experiments

To evaluate the efficacy of the improvements in each part of the network, including the incorporation of the EMA mechanism, the BiFPN architecture, and the WIoU loss function, we conducted ablation experiments that kept the same training strategies and hyperparameters. The performance results can be seen in Table 3, and the complexity results can be seen in Table 4. W represents the AP of crystal wire, R represents the AP of crystal rod, S represents the AP of crystal sheet, and Jlp represents the AP of jelly-like phase. Adding high-resolution features layer P2 was beneficial to improve the AP value of the crystal rod, the crystal sheet, and the crystal wire compared with the original YOLOv8n. Their AP values ranged from 74.2%, 72.2%, and 92.5% up to 76.2%, 74.1%, and 93.4%, respectively. However, utilizing the BiFPN structure resulted in a slight decrease in the AP value of the crystal wire and crystal rod, reducing the complexity of the model and the computational cost. FLOPs dropped by 0.3 G, the parameters came down by 0.14 M, and the model size was reduced by 0.5 MB. The integration of the EMA mechanism before the detection head helped improve the AP value of the crystal rod and crystal sheet. The AP values of the crystal rod and crystal sheet were increased by 1.9% and 1.4%, respectively. Replacing the original SPPF with SimSPPF effectively improved the inference speed while reducing the model size and parameters. Finally, the substitution of the CIoU with WIoU improved the AP values of all the crystals, which improved from 90.1% to 93.3% for the crystal wire, 77.6% for the crystal rod (which was close to the original value), and from 78% to 80.2% for the crystal sheet. Additionally, the value of the recall increased from 81% to 83.8%, which means that the omission of the crystals was alleviated. The increase in precision from 83% to 85.2% signifies an improvement in the accuracy of the model’s predictions. The F1-score is the harmonic mean of precision and recall. A higher F1-score indicates a better balance between precision and recall. The final model has the highest F1 score (84.49%), which makes it the most reliable model for accurate crystal detection.

### 5.3. Comparision of Different Models

The complexity of the model is related to the necessity of applying expensive, high-capacity, high-computing-power equipment. Additionally, the model’s complexity also affects the detection speed. To facilitate a comprehensive comparison, we selected representative models from each category. The two-stage algorithm was Faster-RCNN [40] implemented with a ResNet-50 backbone. For the one-stage algorithm, we chose YOLOv3-tiny, YOLOv5n, YOLOv7-tiny [45], and YOLOv8n. We employed four key metrics to evaluate the complexity of each model, including Floating-Point Operations (FLOPs), the number of parameters, the size of models, and frames per second (FPS). FLOPS represent the number of arithmetic operations required per image; parameters are the total number of parameters that need to be trained and FPS is the metric to evaluate inference speed. The result is shown in Table 5. It is obvious that the complexity of two-stage algorithms was much higher than that of the one-stage algorithms. YOLOv5n had the lowest complexity including FLOPs of 4.1 G, parameters of 1.76 M, and the model size of 3.78 MB. Compared with YOLOv5n, there was an increase in the complexity of YOLOv8n. Although the FLOPs of our method showed a small increase, the parameters and model size were lower, including FLOPs of 11.8 G, parameters of 2.65 M, and a model size of 5.4 MB. The YOLOv7-tiny and YOLOv3-tiny were much more complex than our method. Additionally,, we also compared the detection performance of our method with the above algorithms. We first used AP and mAP metrics to measure the detection accuracy of different algorithms for various indomethacin crystal morphologies. In Table 6, the AP of crystal wire, crystal rod, crystal sheet, and jelly-like phase are 92.5%, 74.2%, 72.2%, and 99.5%, respectively, using YOLOv8n. Although YOLOv8n was not the fastest algorithm, its detection performance exhibited an overall improvement in comparison to the other algorithms while slightly increasing the complexity. Combining detection accuracy and model lightweightedness, we choose YOLOv8n as the best basic model to modify for better detection performance. Firstly, YOLO-PBEWS demonstrated the highest AP values in the detection of each indomethacin crystal morphology. The values were 93.3%, 77.6%, 80.2%, and 99.5% in crystal wire, crystal rod, crystal sheet, and jelly-like phase. Compared with the basic YOLOv8n, there existed an increment of 0.5%, 3.4%, and 8% in crystal wire, crystal rod, and crystal sheet, respectively. Secondly, the precision–recall (PR) curve is a graphical representation used to evaluate the performance of a model, with precision plotted on the vertical axis and recall on the horizontal axis. Precision and recall are inversely related metrics, serving as a measure of a model’s predictive accuracy and its ability to identify all relevant instances, respectively.

### 5.4. Comparision of Different Attention Mechanisms

To augment the model’s capability for feature fusion during detection while maintaining lightweightedness, we integrated the EMA mechanism before the P2 detection head. What seems manifest is that different attention mechanisms lead to different influences on a model’s capability to extract features for the performance of target detection. Therefore, we used the YOLOv8n-P2-BiFPN-SimSPPF-WIoU(YOLO-PBSW) as the baseline and conducted comparative experiments, including adding various prevailing attention mechanisms like CA [50], CBAM [56], SEAM [57], AcMix [58], and EMA. The results are illustrated in Table 7.

The model adopting the CA mechanism had the best value of FPS, reaching up to 79.36. The incorporation of the CBAM attention mechanism into the YOLO-PBSW led to a marginal enhancement in the AP of crystal wire. However, this resulted in a decline in the AP of crystal rod and crystal sheet. The values decreased from 77.2% and 80.6% to 76.3% and 78.4%, which suggests that the CBAM is not an optimal choice for crystal morphology detection. With the addition of the SEAM, there was a 4.5% and 0.3% improvement in the AP of crystal wire and crystal rod, but the AP of the crystal sheet exhibited a 2.9% decline. AcMix caused a significant drop in FPS, which is inadequate for the purpose of real-time detection. The model with the EMA mechanism added exhibited the best detection performance. In each detection, there was an AP value of 93.3% in crystal wire, of 77.6% in crystal rod, of 80.2% in crystal sheet, and of 99.5% in jelly-like phase. Although the AP of crystal sheet slightly decreased compared with the baseline, the AP of crystal wire increased by 4% and the AP of crystal rod increased by 0.4%. Additionally, the model where EMA was added was one of the lightweight models, and FPS could reach 77.52, which illustrated that YOLO-PBEWS achieved a balance between detection performance and speed. This experiment demonstrated the efficacy of integrating the EMA mechanism for recognizing small crystal targets.

**Figure 15:**
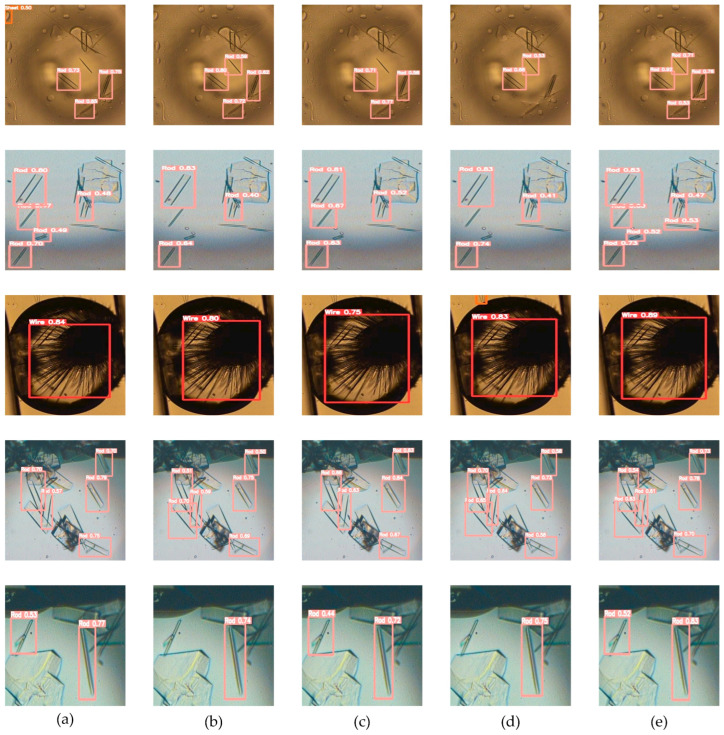
(**a**) Model with AcMix; (**b**) model with CA; (**c**) model with CBAM; (**d**) model with SEAM; and (**e**) model with EMA.

Furthermore, the detection results of adding different attention mechanisms are presented in Figure 15. In the first row, the model where the AcMix mechanism was added incorrectly identified the droplet in the upper-left corner as a crystal sheet. The model where the EMA mechanism was added did not miss the target in the right corner or at the top in comparison with the models with SEAM and CBAM. In the second line, it is obvious that the performance of the model with the EMA mechanism was the best. Models incorporating other attention mechanisms exhibited a range of degrees of target missing. In the third row, compared with other attention mechanisms, the model with the EMA mechanism showed the most outstanding performance in detecting crystal wire. In the fourth line, although the model fused with CA and SEAM identified as many crystal rods as the model merged with EMA, it demonstrated greater confidence. In the fifth line, the model with the EMA mechanism added was able to recognize the remaining crystal rod in the image. In sum, the model with EMA showed excellent performance in the detection of different crystal morphologies.

### 5.5. Comparision of Different Loss Functions

To assess the effectiveness of different loss functions in enhancing the model for our dataset, we conducted the experiment with different loss functions like CIoU, MPDIoU, EIoU, ShapeIoU, and WIoUv3. In addition, we selected YOLOv8n-BiFPN-EMA-SimSPPF (YOLO-PBES) as the base model.

What seems manifest from Table 8 is that the model adopting WIoUv3 demonstrated superior performance in terms of precision, recall, mAP, and FPS compared to models using other loss functions. High precision indicates that when the model predicts a positive instance, it is highly likely to be correct, and high recall suggests that the model is effective at minimizing false negatives, ensuring that fewer instances of the target class are missed during detection. The model with WIoUv3 reached 85.2% and 83.8%, which was better than the others. Additionally, it is noteworthy that the values of mAP and FPS were 87.6% and 77.52, respectively. In comparison to the model applicating CIoU, which was the original loss function, utilizing WIoUv3 increased the loss function by 1.1% and 14.63%, respectively, which means that the WIoUv3 loss function significantly enhanced both the model’s detection performance and speed.

### 5.6. Comparision of Different Improved Models

FFCA-YOLO is an enhanced version of the YOLO model specifically designed for the improved detection of small objects in remote sensing images. To further validate the effectiveness of the proposed method, we introduced FFCA-YOLO and its lightweight model, L-FFCA-YOLO, for comparison.

As shown in Table 9, YOLO-PBESW is the most efficient model, with the lowest FLOPs (11.8 G), the smallest size (5.46 MB), and the highest FPS (77.52), making it ideal for real-time applications. Firstly, for the more challenging small-object-detection task, our model demonstrated an advantage in detecting crystal rod and crystal sheet, achieving AP improvements of 4.6% and 5.4% over FFCA-YOLO, as well as 3.9% and 7.4% over L-FFCA-YOLO, respectively (Table 10). Secondly, Table 11 shows that YOLO-PBEWS outperforms the other models, with a leading mAP of 87.6%. It is 3.4% higher than FFCA-YOLO and 3.8% higher than L-FFCA-YOLO. Additionally, the YOLO-PBESW model achieved the highest F1-score of 84.49%, suggesting that YOLO-PBESW is more effective in maintaining a low rate of false positives and false negatives.

**Figure 16:**
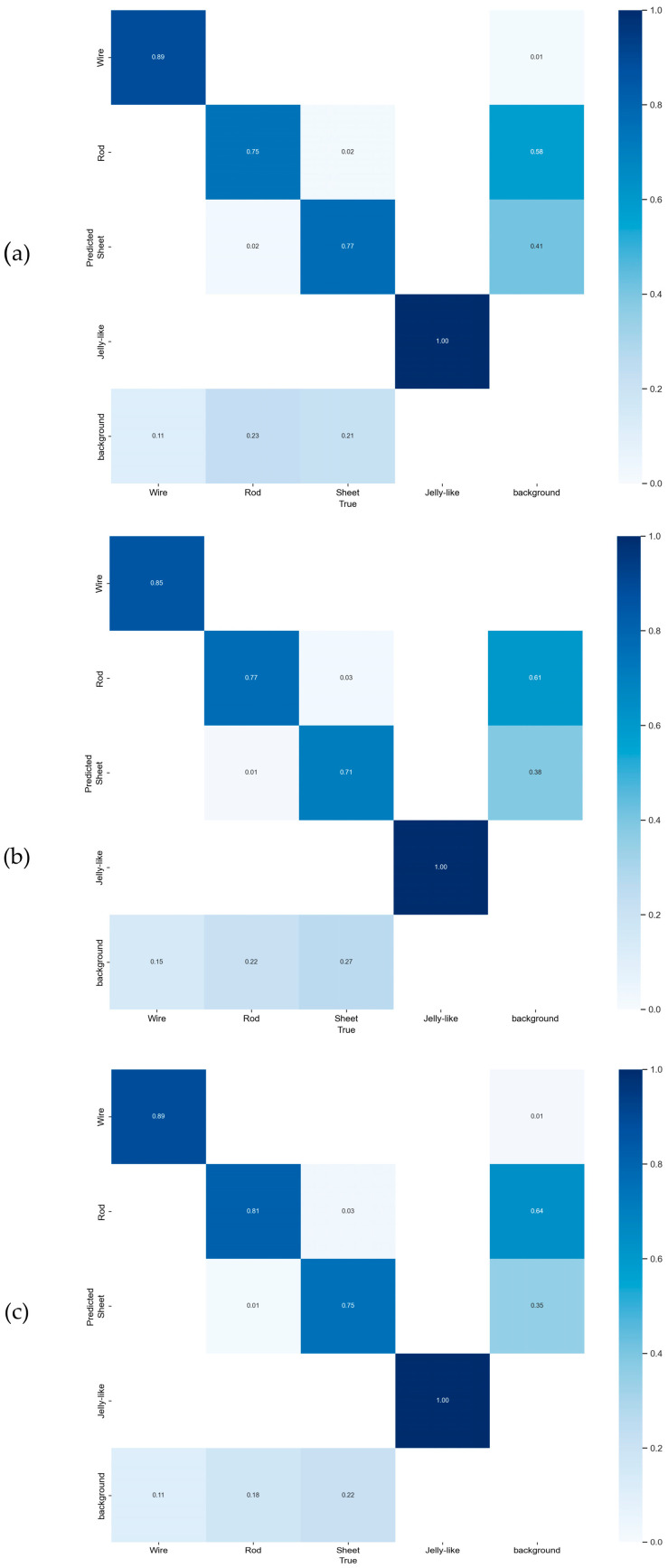
Confusion matrices of different improved models: (**a**) FFCA-YOLO, (**b**) L-FFCA-YOLO, and (**c**) YOLO-PBESW. In each matrix, the rows represent the actual classes, while the columns represent the predicted classes. The diagonal elements indicate the correct classifications, with darker shades representing higher accuracy.

Based on the comparison of confusion matrices (Figure 16), YOLO-PBESW outperformed the other models in detecting crystal sheet and crystal rod morphologies, achieving 80.2% accuracy for crystal sheet and 77.6% for crystal rod. In comparison, FFCA-YOLO reached only 74.8% accuracy for crystal sheet and 73% for crystal rod, while L-FFCA-YOLO lags further behind with 72.8% for crystal sheet and 73.7% for crystal rod. These results demonstrate YOLO-PBESW’s superior ability to accurately distinguish between crystal sheet and crystal rod morphologies, leading to lower misclassification rates and more dependable detection outcomes.

**Figure 17:**
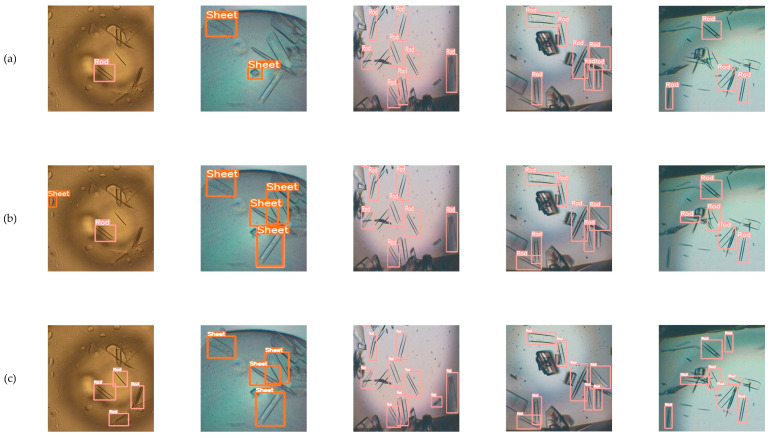
(**a**) FFCA-YOLO; (**b**) L-FFCA-YOLO; and (**c**) YOLO-PBESW.

Figure 17 illustrates the detection comparison of YOLO-PBESW with two other small target detection models. In the first column, FFCA-YOLO missed several instances, and L-FFCA-YOLO incorrectly identified the upper left droplet as a crystal sheet. In the third set of images, only YOLO-PBEWS recognized the crystal rod in the bottom right corner. In the last column, our model identified the largest number of targets. This result shows YOLO-PBEWS has better detection performance.

## 6. Conclusions

The bioavailability, solubility, permeability, and other properties of drugs are primarily influenced by the crystal morphology. Different drug crystals require specific crystallization conditions. This paper focused on indomethacin and proposed the YOLO-PBESW network to alleviate the issue of poor recognition and slow detection of indomethacin crystal morphologies, including crystal wire, crystal rod, crystal sheet, and jelly-like phase, thereby reducing the consumption of reagents and active pharmaceutical ingredients. We utilized a high-throughput droplet microfluidic system to generate indomethacin crystals, which were captured using a CCD camera and imaging software. The model enhanced the detection efficacy of small targets by integrating a high-resolution feature layer P2, and adopted the concept of BiFPN to modify network structure for reducing the computational cost and increasing the feature extraction. Additionally, we only added the EMA mechanism before the P2 detection head for improving the attention of networks to global features while maintaining lightweightedness. Furthermore, we substituted SimSPPF, which adopts the ReLU activation function, with SPPF, to lower the computational costs. Finally, the CIoU loss function was substituted with the WIoUv3, which utilized a dynamic non-monotonic focus mechanism to improve the performance of detection.

The experimental results demonstrated that the YOLO-PBESW network could reach 87.6% in the mAP metric. In addition, the model size and FPS were 5.46 MB and 77.52, respectively. Our proposed method demonstrates a superior overall performance compared to established algorithms, including Faster-RCNN, YOLOv3-tiny, YOLOv5n, and YOLOv7-tiny, when evaluated for small-target detection on crystals within high-throughput screening droplets. In addition, compared to other algorithms designed for small-object detection, such as FFCA and its lightweight version L-FFCA, our model exhibits lower complexity while achieving better performance in terms of reducing both false negatives and false positives. This exceptional performance suggests significant promise for this method in the study of crystallization. By integrating YOLO-PBEWS with droplet microfluidics technology, we can rapidly identify and classify crystal wire, crystal rod, crystal sheet, and jelly-like phase. This combination enables the precise determination of crystallization conditions for the three types of crystals and helps to avoid conditions that lead to jelly-like formation, thereby significantly reducing the consumption of reagents and active pharmaceutical ingredients.

The shortcomings of this work and future research directions are as follows. Firstly, the dataset used in this study was not sufficiently comprehensive. In future studies, the dataset will be augmented and enhanced by acquiring a larger number of videos and images for model training and testing. Secondly, the model proposed in this work has been validated only for the recognition of indomethacin crystal morphologies and has not yet been tested on other drug crystal morphologies. Future work will focus on validating and optimizing the model for the recognition of other drug crystals. Thirdly, although the model we proposed demonstrates generally effective performance, its accuracy can be compromised when crystals are partially occluded. This issue may affect detection in scenarios where crystals are not fully visible. We will explore techniques such as distillation and network pruning in the future to further reduce computational complexity and enhance detection performance. Furthermore, we intend to adapt the model for deployment on edge computing platforms. This will involve optimizing the algorithm for reduced computational complexity and memory footprint to ensure seamless integration and operation within such platforms.

## Figures and Tables

**Table 1:** Experiments’ environment configuration.

Experimental Component	Version
Operational System	Windows 10
CPU	Intel Xeno Sliver 4210R
GPU	RTX5000
CUDA version	11.8
Python version	3.8
Pytorch version	2.0.1

**Table 2:** Training setting.

Parameter	Value
Epochs	300
Batch Size	12
Gradient-based optimizers	SGD
Initial learning rate (lr0)	0.01
Momentum	0.937
Weight decay	0.0005

**Table 3:** Ablation experiments 1. The ‘√’ symbol indicates that the corresponding technique was utilized in the model’s construction for detection.

YOLOv8	P2	BiFPN	EMA	SimSPPF	WIou	W (%)	R (%)	S (%)	Jlp (%)	Precision (%)	Recall (%)	F1 (%)	mAP@0.5 (%)
√						92.5	74.2	72.2	99.5	83.4	80.6	81.9	84.6
√	√					93.4	76.2	74.1	99.5	84.7	82.2	83.4	85.8
√	√	√				92.3	75.7	76.3	99.5	84.5	80.5	82.4	86
√	√	√	√			88.7	77.6	77.7	99.5	83.4	81.4	82.4	85.9
√	√	√	√	√		90.1	78.5	78	99.5	83	81	81.9	86.5
√	√	√	√	√	√	93.3	77.6	80.2	99.5	85.2	83.8	84.5	87.6

**Table 4:** Ablation Experiments 2. The ‘√’ symbol indicates that the corresponding technique was utilized in the model’s construction for detection.

YOLOv8n	P2	BiFPN	EMA	SimSPPF	WIou	FLOPs (G)	Parameters (M)	Size (MB)	FPS
√						8.1	3	6.1	119
√	√					12.2	2.92	5.96	96.15
√	√	√				11.9	2.78	5.46	88.5
√	√	√	√			11.8	2.67	5.69	76.9
√	√	√	√	√		11.8	2.66	5.46	79.3
√	√	√	√	√	√	11.8	2.65	5.46	77.52

**Table 5:** Comparison of complexity.

Model	FLOPs (G)	Parameters (M)	Size (MB)	FPS
Faster-RCNN	370.2	137	108	7.46
YOLOv3-tiny	18.9	12.1	23.2	166.7
YOLOv5n	4.1	1.76	3.78	111.1
YOLOv7-tiny	13.0	6.01	12	96.15
YOLOv8n	8.1	3	6.1	119
L-FFCA-YOLO	3.8	0.47	1.85	62.89
YOLO-PBESW	11.8	2.65	5.46	77.52

**Table 6:** Comparison of performance.

Model	Crystal Wire AP	Crystal Rod AP	Crystal Sheet AP	Jelly-like Phase AP	mAP
Faster-RCNN	93%	23%	25%	100%	68.35%
YOLOv3-tiny	87.3%	66%	64.5%	99.5%	84.6%
YOLOv5n	87.2%	69.5%	69.3%	99.5%	81.4%
YOLOv7-tiny	89.4%	71.4%	72.9%	99.7%	83.4%
YOLOv8n	92.5%	74.2%	72.2%	99.5%	84.6%
L-FFCA-YOLO	88.8%	71%	70.4%	99.1%	82.3%
YOLO-PBESW	93.3%	77.6	80.2%	99.5%	87.6%

**Table 7:** Comparison of different attention mechanisms.

Model	Crystal Wire AP	Crystal Rod AP	Crystal Sheet AP	Jelly-like Phase AP	Model Size	FPS
YOLO-PBSW	89.3%	77.2%	80.6%	99.5%	5.46 MB	78.13
+CA	91.7%	78.1%	79.5%	99.5%	5.46 MB	79.36
+CBAM	89.9%	76.3%	78.4%	99.5%	5.46 MB	72.46
+SEAM	93.8%	77.9%	78.8%	99.5%	5.47 MB	75.18
+AcMix	89%	77.5%	77.7%	99.5%	5.48 MB	57.80
+EMA	93.3%	77.6	80.2%	99.5%	5.46 MB	77.52

**Table 8:** Comparison of different loss functions.

YOLO-PBES	Precision (%)	Recall (%)	mAP@0.5 (%)	FPS
CIoU	83%	81%	86.5%	62.89
MPDIoU	82%	82.2%	86%	73.53
EIoU	83.9%	82.1%	86.1%	74.63
ShapeIoU	84.4%	80.7%	85.3%	76.34
WIoUv3	85.2%	83.8%	87.6%	77.52

**Table 9:** Comparison of different improved models.

Model	FLOPs (G)	Parameters (M)	Size (MB)	FPS
FFCA-YOLO	51.2	7.1	14.5	58.13
L-FFCA-YOLO	37.1	5.04	10.6	50.25
YOLO-PBESW	11.8	2.65	5.46	77.52

**Table 10:** Comparison of improved models’ performance 1.

Model	Crystal Wire AP	Crystal Rod AP	Crystal Sheet AP	Jelly-like Phase AP
FFCA-YOLO	89.6%	73%	74.8%	99.5%
L-FFCA-YOLO	89.4%	73.7%	72.8%	99.3%
YOLO-PBESW	93.3%	77.6	80.2%	99.5%

**Table 11:** Comparison of improved models’ performance 2.

YOLO-PBES	Precision (%)	Recall (%)	F1-Score (%)	mAP@0.5 (%)
FFCA-YOLO	85.6	80.7	83	84.2
L-FFCA-YOLO	86	81.4	83.6	83.8
YOLO-PBESW	85.2	83.8	84.5	87.6

## Data Availability

The data supporting this study’s findings can be obtained from the corresponding author upon reasonable request. However, the data are not publicly available due to privacy concerns.
